# The promising future of microalgae: current status, challenges, and optimization of a sustainable and renewable industry for biofuels, feed, and other products

**DOI:** 10.1186/s12934-018-0879-x

**Published:** 2018-03-05

**Authors:** Muhammad Imran Khan, Jin Hyuk Shin, Jong Deog Kim

**Affiliations:** 1 Department of Biotechnology, Chonnam National University, San 96-1, Dun-Duk Dong, Yeosu, Chonnam 550-749 South Korea; 2 Research Center on Anti-Obesity and Health Care, Chonnam National University, San 96-1, Dun-Duk Dong, Yeosu, Chonnam 550-749 South Korea

**Keywords:** Microalgae, Biofuels, Carbon dioxide mitigation, Viable biomass, Culture parameters, Pretreatment, Bioactive compounds

## Abstract

Microalgae have recently attracted considerable interest worldwide, due to their extensive application potential in the renewable energy, biopharmaceutical, and nutraceutical industries. Microalgae are renewable, sustainable, and economical sources of biofuels, bioactive medicinal products, and food ingredients. Several microalgae species have been investigated for their potential as value-added products with remarkable pharmacological and biological qualities. As biofuels, they are a perfect substitute to liquid fossil fuels with respect to cost, renewability, and environmental concerns. Microalgae have a significant ability to convert atmospheric CO_2_ to useful products such as carbohydrates, lipids, and other bioactive metabolites. Although microalgae are feasible sources for bioenergy and biopharmaceuticals in general, some limitations and challenges remain, which must be overcome to upgrade the technology from pilot-phase to industrial level. The most challenging and crucial issues are enhancing microalgae growth rate and product synthesis, dewatering algae culture for biomass production, pretreating biomass, and optimizing the fermentation process in case of algal bioethanol production. The present review describes the advantages of microalgae for the production of biofuels and various bioactive compounds and discusses culturing parameters.

## Introduction

Algae are photosynthetic organisms that grow in a range of aquatic habitats, including lakes, pounds, rivers, oceans, and even wastewater. They can tolerate a wide range of temperatures, salinities, and pH values; different light intensities; and conditions in reservoirs or deserts and can grow alone or in symbiosis with other organisms [1]. Algae are broadly classified as Rhodophyta (red algae), Phaeophyta (brown algae), and Chlorophyta (green algae) and classified by size as macroalgae or microalgae. Macroalgae (seaweed) are multicellular, large-size algae, visible with the naked eye, while microalgae are microscopic single cells and may be prokaryotic, similar to cyanobacteria (Chloroxybacteria), or eukaryotic, similar to green algae (Chlorophyta).

**Fig. 1:**
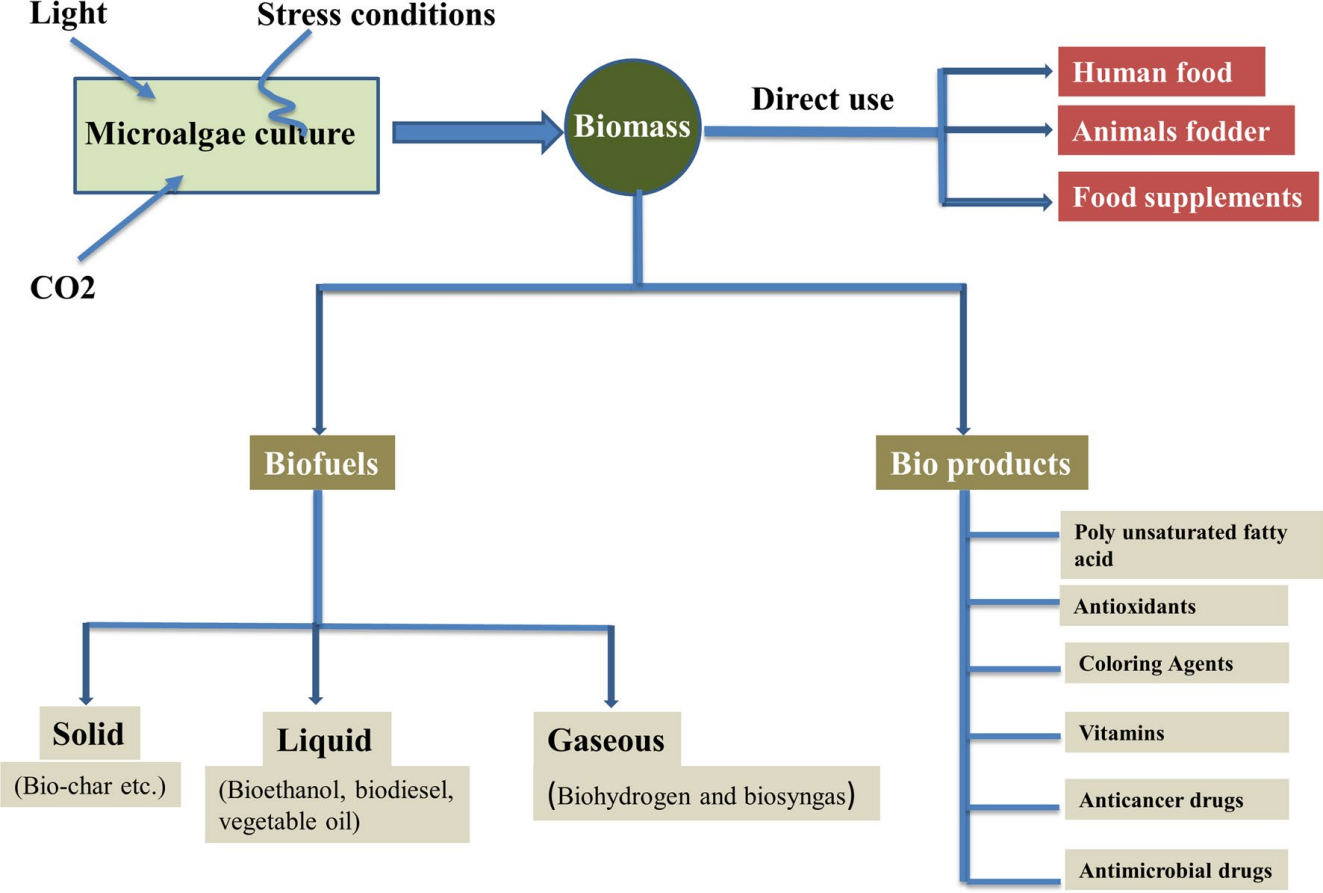
Microalgae convert atmospheric CO_2_ to carbohydrates, lipids, and other valuable bioproducts by using light. Microalgae biomass is a rich source for biofuels and bioactive compounds

Microalgae can be a rich source of carbon compounds, which can be utilized in biofuels, health supplements, pharmaceuticals, and cosmetics [2]. They also have applications in wastewater treatment and atmospheric CO_2_ mitigation. Microalgae produce a wide range of bioproducts, including polysaccharides, lipids, pigments, proteins, vitamins, bioactive compounds, and antioxidants (Fig. 1) [3]. The interest in microalgae as a renewable and sustainable feedstock for biofuels production has inspired a new focus in biorefinery. Growth enhancement techniques and genetic engineering may be used to improve their potential as a future source of renewable bioproducts.

The industrial cultivation of microalgae to produce biofuels and bioproducts has increased dramatically over the last few decades [4]. Algae are produced in quantity and sold directly as food and nutrient supplements, while their processed products or extracts are used in biopharmaceuticals and cosmetics [5–7].

### Bioenergy and microalgae

The rapid growing population of the world continuously increases the global demand for fuel energy. The intensive use of fossil fuels worldwide leads to its depletion and will bring them close to the point of exhaustion due to unsustainable and nonrenewable nature. Thus, biofuels are now a growing opportunity throughout the world as alternative to fossil fuels. Some developed countries are already producing biofuels at the commercial level. Biofuels such as biodiesel and bioethanol are proving to be excellent alternative fuels and can be produced from several resources of biomass, such as food crops, crop wastes or fruits, woody parts of plants, garbage, and algae [8, 9]. The advantageous features of biofuels produced from biomass are renewability and a significantly smaller contribution to environmental pollution and global warming. The emission of greenhouse gases mainly CO_2_ from burning of fossil fuels are the main cause of global warming. Fossil fuels are responsible for 29 gigatons/year release of CO_2_ with a total of 35.3 billion tons CO_2_ till now [10]. Biofuels including algal fuels have oxygen levels of 10–45% and very low levels of sulphur emission while petroleum-based fuels have no oxygen levels with high sulphur emission. Biofuels are non-polluting, locally available, accessible, sustainable and reliable fuel obtained from renewable sources. Microalgae algae-based fuels are ecofriendly, nontoxic and with strong potential of fixing global CO_2_. It has been reported that 1 kg of algal biomass is can fix 1.83 kg of CO_2_ furthermore some species use SOx and NOx as nutrient flow along with CO_2_ [11]. CO_2_ constitutes 50% of dry weight of algal biomass. The selection and development of biomass is a crucial, cost-limiting phase in biofuels generation for adjusting and optimizing energy structure and cost. Selection of biomass for biofuels production is also directly related to greenhouse gas emissions, environmental and economic sustainability [12]. The current focus is on microalgae as a feedstock for bioenergy production as the most promising raw material to compensate and balance the ever-increasing demands for biofuels, food, feed and valuable chemicals production [9, 10]. Many countries in Asia, Europe, and America have started industrialization of bioenergy from microalgae biomass.

Microalgae are rapidly growing photosynthetic organisms having potential of transforming 9–10% of solar energy (average sunlight irradiance) into biomass with a theoretical yield of about 77 g/biomass/m^2^/day which is about 280 ton/ha/year [13, 14]. At lager scale cultivation this yield is lower both in outdoor and indoor culture system. In Photobioreactors the actual yield is lower due to loss of absorbed active radiation [15–17], Proper shaking and mixing of the culture in the bioreactor is necessary for uniform distribution of light energy to avail the same strength to all the cells to convert maximum light energy to biomass.

In several aspects, microalgae feedstock is competent and preferable to produce biofuels for examples microalgae don not require cultivable land and fresh water for cultivation, they are not edible therefore no effect on human and animal’s food chain, can be grown to serval folds irrespective to seasonal conditions, mitigation of atmospheric CO_2_ and treatment of waste water [18, 19]. Absence of lignocellulosic materials in microalgae cell wall facilitate the pretreatment process and reduce overall cost of production. Microalgae can feed on industrial wastes and the processing energy is less than the energy produced by the algae [20–22]. Second generation biofuels involve terrestrial plants, especially food crops as feedstocks, a highly controversial issue, since biofuels production from food crops can only occur at the expense of their use as food and feed. Additionally, crop foods require arable land and large amounts of water, which makes their use for fuel production unsustainable and thus, incomputable as alternative liquid fuels [23, 24]. The algal fuels technology is still incipient, and much improvement is required to make it commercially attractive to both, investors and consumers.

Most of the microalgae species are favorable for biodiesel production due to high lipids contents 50–70% and may reach to 80% such as in case of the microlaga *B. braunii* which accumulate up to 80% of oil in it biomass [25–27]. Microalgae are capable of producing algal oil 58,700 L/hac which can produce 121,104 L/hac biodiesels [11, 28, 29]. The infeasibility of algal biodiesel is due to the associated high operational, maintenance, harvesting and conversion cost [30].

Bioethanol is one of the major and clean biofuel used as transportation fuel. Bioethanol has many advantages over fossil fuels, such as (i) high octane number in bioethanol prevents knocking of cylinders in engines (ii) due to the presence of higher oxygen contents, bioethanol burning produces much less greenhouse-effect gasses (iii) Bioethanol is the only biofuel that can be used directly in the current automotive industry without any modifications (iv) Bioethanol can be mixed with oil [31–33]. Global production of biofuels has increased from 4.8 to 16.0 billion of gallons from 2000 to 2007 [34]. Currently, the USA and Brazil are the world leaders in the production of bioethanol. Their contribution is approximately 75–80% of the world total bioethanol production [32, 34]. The USA have 187 commercial bioethanol plants, which mainly produce bioethanol from corn grain [31]. In 2013, Brazil produced 37 billion l of bioethanol using sugar cane as the main feedstock. The European Union (EU), uses wheat and sugar beet as the preferred feedstock for bioethanol production and produces 2.0 billion gallons annually [31, 32].

Biofuels from renewable and sustainable feedstock are the future permissive energy sources in place of fossil liquid fuels. Today, bioethanol is the most common biofuel, mainly produced from sugars of corn and sugarcane, but the technology is now shifting towards algal carbohydrates as potential raw materials for bioethanol production [35–37]. Global bioethanol production has vigorously increased from 1 billion to 39 billion l within a few years and will reach 100  billion l soon [38].

**Fig. 2:**
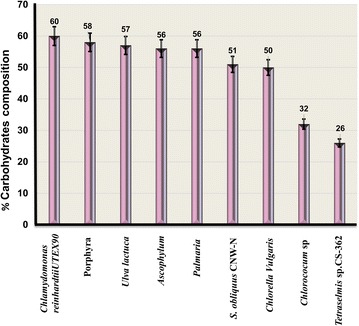
Different species of carbohydrate-rich microalgae that compose feasible feedstock for bioethanol production

Microalgae possess high contents of different carbohydrates, such as glycogen, starch, agar and cellulose, etc. which can be easily converted to fermentable sugars for bioethanol production [39]. Figure 2 presents carbohydrate-rich microalgae species suitable for algal bioethanol production.

Extraction of the stored carbohydrates from algae cells needs lysis of the cells. This may be accomplished in different ways, such as enzymatic, acidic or solvent extraction. However, ethanol yields depend on the method followed for the extraction. The extracted sugars need pretreatment to break the polymer molecules to monomeric forms that can be subjected to microbial fermentation to yield bioethanol [8].

Bioethanol production from microlage is an excellent effort in the development of sustainable biofuels, still there are some challenges regarding large-scale production and commercialization of this clean biofuel. The main areas in the development of algal bioethanol technology, which should be optimized for the commercialization of bioethanol are, selection of the algal biomass, pretreatment and an efficient fermentation process. Fermentative ethanol yield greatly depends on the potential of the fermenting microorganisms used. The fermentation process should be carried out in an aseptic environment to avoid contamination, which greatly reduces final yields [40]. The potential of microalgae to become a competitive feedstock for bioethanol production will require a continued effort to overcome current limitations regarding (i) culturing algae and producing carbohydrate- rich biomass (ii) dewatering and harvesting (iii) pretreatment of biomass (iv) ensuring maximum yielding fermentation. Development of a cost-effective algal system can be achieved by improvement and optimization of each of the above mentioned. Both biomass and carbohydrate productivity of algal cells need to be increased for economical and feasible production of bioethanol [7]. Some carbohydrates rich microalgae like *Chlamydomonas reinhardtii* and *Chlorella vulgaris* are considered to be potential for techno-economic analysis (TEA) of bioethanol production [41]. TEA of commercial bioethanol production from microalgae estimate suitability of the plant with respect to total investment, total cost ant total net profit [42].

Increasing the feasibility of microalgae for higher bioethanol yields through fermentative production, requires careful consideration of several parameters. The key factor for economic feasibility of microalgae biofuels are maximizing the algal biomass with reduction of operational and maintenance cost [43]. It has been stated that the economic viability of algal fuels need at least more 10 years research and development to achieve a stable position [44]. US Renewable Fuels Standard (RFS) estimated to obtain 36 billion gallons of microalgae-based fuels by the year 2022 [45].

Microalgae based fuels are estimated to be economical for crude petroleum sell higher than 100 USD per barrel [46]. Although the algal biofuels are not yet economically feasible, there are many companies in USA, Europe and other regions of the word that are producing algal fuels at commercial scale [11]. According to the TEA model biodiesel from algal biomass below $5/gallon gasoline equivalent and bioethanol at the cost $2.95/gal are economically viable [47]. Some studies suggested the feasible and economically viable price of algae competitive to other biofuels is $1/L [18]. Several companies, e.g. Algenol, Sapphire Energy and Seambiotic etc. are involved in commercial scale production of bioethanol with output 1 billion gallons/year costing at 85 cents/l [48].

### Algae culturing

Large-scale microalgae cultivation will decisively contribute to the development of a sustainable industry for biomass production as well as generating cost-effective high-value products. Many species of microalgae show potential for large-scale cultivation, but there is insufficient information to run commercial trials. A huge amount of microalgae biomass is required to compete with other feedstocks for sustainable production of bioethanol. Successful microalgae culturing technologies will need to create larger amounts of biomass, which will make the use of food stuffs for bioethanol production comparatively less attractive.

Microalgae can be cultured by different methods and under different conditions. They need light as an energy source to convert the absorbed water and CO_2_ into biomass through photosynthesis [49]. Photosynthetic products accumulate in various forms, such as cell components or storage materials, and vary from 20 to 50% of total biomass [25]. Algae also need nitrogen and phosphorus as major nutrients, which account for 10–20% of algae biomass [50]. Other requirements for growth are the macronutrients Na, Mg, Ca, and K; micronutrients, such as Mo, Mn, B, Co, Fe, and Zn; and other trace elements. Wastewater is a good source of the required nutrients for microalgae cultivation. Thus, application of organic effluents from the food and agriculture industries can nourish microalgae.

During growth, the algae cells pass through different phases (e.g., lag, exponential, stationary, death). Different species of microalgae may vary in their need for growth media. However, the major requirements are the same for almost all species and include essential nutrients, an organic or inorganic carbon source, nitrogen, phosphorus, and iron [51].

One of the most important parameters in algae culturing is the type of bioreactor used. This should be designed according to the species and the purpose of culture. On a large scale, algae can be cultured in open ponds (high-rate ponds). Open culture systems are comparatively inexpensive, but they become easily contaminated. Other bioreactors have continuous or batch culture facilities [52, 53]. Some species of algae grow very well in heterotrophic culture [54]. For commercial cultivation it is feasible to grow microalgae in waste water treatment plant to get dual advantages of water treatment and biomass production. See water is also a good alternative for commercial cultivation of microalgae. Using see water instead of fresh water for microalgae culturing will reduce the cost of production. Marin water is a good media for microalgae culture avoiding nutrients cost and enhancing productivity of lipids and other useful products in microalgae biomass [55]. Most recently ocean cultivation system has attracted attention for commercial scale production of algae due the advantages like mixing of the culture by ocean waves, utilizing dissolve nutrients large area availability, etc. which result in the reduction of culturing and maintenance cost [55–57].

To make the algae biotechnology sustainable, feasible and economically viable it is necessary to develop successful culturing technologies for targeted production of biomass. For a feasible algal culture, the biomass output should be > 30 g/m^2^-day [58]. Approximately 40,000 different species of microalgae have been reported [59]. Microalgae culture depends on the species and is affected by factors such as nutrient availability (N, P, K, etc.), temperature, pH, salinity, inorganic carbon, oxygen, light intensity, and CO_2_ [60]. Other important factors that determine the success of culture include stirring and mixing, width and depth of the bioreactor, harvest frequency, and dilution rate. Following are the important parameters of culture which has great influence and impact on the overall yield of biomass and bioproducts in microalgae.

#### Light

Light intensity is one of the major limiting factors in microalgae cultivation. Light duration and intensity directly affect photosynthesis of microalgae and has influence on the biochemical composition of microalgae and biomass yield [61]. In modeling of the outdoor or indoor algal culture system, growth rate and biomass productivity are predicted as a function of light [62]. Light intensities vary inside the culture and reduce in culture depth this should be taking in consideration for modeling of the bioreactor or open pond system. Algae species vary in terms of their light requirements for maximum growth and biomass accumulation.

At very low and very high light intensities, microalgae cannot grow efficiently [60, 63–65]. At the compensation point, where photosynthetic CO_2_ uptake exactly matches respiratory CO_2_ release, net growth is zero. Higher light intensities will increase photosynthetic rate to some maximum point, after which it levels off until the photosynthetic rate is balanced by photorespiration and photoinhibition. Thus, optimal light intensity needs to be determined experimentally in each case to maximize CO_2_ assimilation with a minimum rate of photorespiration and as little photoinhibition as possible [65]. A specific duration of light/dark periods is required for algal photosynthesis. Light is required for synthesis of ATP and NADPH, which drive the dark reactions of photosynthesis that produce carbon skeletons [66]. There is a direct relationship between microalgae growth and light intensity and duration, up to the saturation point. Khoeyi et al. [67] experimentally proved the differences in the growth rate and biomass yield by growing the same algae strain under different light intensities and for various durations. The growth rate and biomass productivity decreased with decreasing light duration [68]. Most studies have shown that 16 h light/8 h dark is most suitable for algae growth. Appropriate light intensity and duration is necessary in bioreactors for microalgae to avoid photo-oxidation and growth inhibition [69]. Appropriate penetration and uniform distribution of light is also needed to avoid photoinhibition, also called the self-shading effect, in which algae at lower layers are shaded from the light by upper layers. LED lights are a good choice for this purpose, although fluorescent tubes can also be used [70]. Mata et al. [60] reported that an aerated culture of microalgae under 12,000 lx intensities for 12 h of daylight produced a higher biomass yield, whereas biomass decreased when the light intensity was reduced. Khan et al. [71] reported that *microcystis aeruginosa* give maximum biomass and carbohydrates productivity at red LED light of about 5000 lx. Daliry et al. most recently reported the maximum growth rate and lipid production by *Chlorella vulgaris* at light intensities of 5000–7000 lx [72]. The optimum level of light intensities for most of the microalgae species are about 200–400 μM photons/m^2^/s [73]. Photoinhibition can be prevented by increasing the light intensity or by thoroughly mixing the culture continuously. Hence, light directly affects the final yield of biomass and synthesis and accumulation of carbohydrates in the algal cells. Kitaya et al. [74] experimentally proved that a light intensity of 100 µmol/m^2^/s is optimum for some microalgal species.

#### Temperature

Temperature is another important factor in the growth of microalgae and directly influences the biochemical processes, including photosynthesis, in the algal cell factory. Each species has its own optimal growth temperature. Increasing temperature to the optimum range exponentially increases algal growth, but an increase or decrease in the temperature beyond the optimal point retards or even stops algae growth and activity [75]. The optimum temperature range for most algal species is 20–30 °C [76] although thermophile algae such as *Anacystis nidulans* and *Chaetoceros* can endure temperatures up to 40 °C and algae growing in hot spring near temperature 80 °C [77]. Growing microalgae cultures at non-optimal temperatures will result in high biomass losses, particularly in outdoor culture systems [63, 78, 79]. Temperature is important factor for large scale cultivation specially in open pond culture and need careful monitoring as the algae experience significant temperature change over time [80].

Low temperatures affect photosynthesis by reducing carbon assimilation activity, whereas too-high temperatures reduce photosynthesis by inactivating the photosynthetic proteins and disturbing the balance of energy in the cell. Higher temperature also reduces cell size and respiration. The decline in photosynthesis results in a decreased growth rate [81]. The key effect of temperature on photosynthesis is due to a decline in the activity of ribulose-1,5-bisphosphate (Rubisco), an enzyme with dual functions. It can act as an oxygenase or as a carboxylase, depending on the relative amounts of O_2_ and CO_2_ present in the chloroplasts. CO_2_ fixation activity of Rubisco enzyme increases with rising temperature up to a certain level and then declines [82]. Hence, temperature is a limiting factor for algal growth rate and biomass production through its influence on the affinity of ribulose for CO_2_.

Temperature can also be used as a stress treatment to induce the production of valuable metabolites [83]. Converti et al. [84] found that a culture of Chlorella vulgaris produced more carbohydrates and lipids if grown at 25 °C than at 30 °C. Kitaya et al. [74] found that temperatures between 27 and 31 °C were optimum for several microalgae species.

#### Nutrients

Different microalgae species may vary in their nutritional needs; however, the basic requirements are same for all species. Nitrogen, phosphorus, and carbon form the backbone of microalgae (CH_1.7_ O_0.4_ N_0.15_ P_0.0094_) [85] and are classified as macronutrients required for algal growth. Some marine microalgae species also require silicon as a macronutrient. Microalgae absorb O_2_ and H_2_ from water. The quantities of macronutrients such as nitrogen and phosphorus may vary for different species of microalgae. It has been reported the growth of *chlorella* declined when the concentrations of nitrogen and phosphors reduced from 31.5 and 10.5 mg/l respectively [86]. Quantities of the available nitrogen in the culture directly alter cell growth. Nitrogen limitation in the microalgae culture, can reduce growth and biomass productivity although they increase production of carbohydrates and lipids. 0.5 g/l nitrogen has been proved to be optimum concentration for *Chlorella vulgaris* at which it produces 3.43 g/l biomass [72].

the micronutrients Mo, K, Co, Fe, Mg, Mn, B, and Zn are only required in trace amounts but have a strong impact on microalgae growth, as they influence many enzymatic activities in algal cells [78, 87] Usually, inorganic nitrogen and phosphorus are absorbed as nitrates and phosphates. Urea is also a suitable source and a cost-effective alternative to other inorganic nitrogen sources. Carbon can be added to the algae culture in organic forms, such as glycerol or acetates, or as CO_2_. However, for large-scale cultivation of microalgae, environmental CO_2_ must be used as a carbon source, which is not only low cost but adds the benefit of CO_2_ mitigation. P, N, and C are the primary inorganic nutrients that are essential for microalgal growth [88]. Nutrient deficiency greatly affects the microalgae growth rate and results in low biomass [89–92]. The nutrient supply strongly affects the synthesis and accumulation of carbohydrates and lipids in microalgae [91].

For commercial production of microalgae biomass, the culture must be grown rapidly; thus, providing the proper nutrients is very important to speed algal growth. Some strongly limiting substances can be used as growth enhancers for microalgae. In addition, certain bacteria can enhance the growth rates of microalgae by supplying important nutrients. These bacteria degrade nutrients into forms that can be readily assimilated by microalgae, such as ammonia or nitrate [93].

#### Mixing

Mixing and aerating provide uniform distribution of nutrients, air, and CO_2_ in microalgae culture. They also enable the penetration and uniform distribution of light inside the culture and prevent the biomass from settling and causing aggregation [94]. If all the other requirements are met but there is no mixing, biomass productivity will be lowered significantly. Thus, microalgae cultures must be continuously mixed to keep all cells in suspension with free access to light. A proper mixing system in a photo-bioreactor not only enables nutrient dissolution and light penetration into the culture but also provides for efficient gaseous exchange [95].

#### pH and salinity

The pH of the culture media is another important factor affecting microalgae growth. Microalgae species have different pH requirements. Most grow well in the pH range from 6 to 8.76 Different sources of growth media have different pH values. Most algae species are pH sensitive and few can endure a range of pH as broad as that tolerated by *C. vulgaris* [96]. *C. vulgaris* can grow in broad range of pH however the maximum growth rate and biomass productivities are reported at pH 9–10 [72]. Increasing the pH will increase the salinity of the culture media, which is very harmful for algae cells [85].

#### Mixotrophic cultivation

In autotrophic cultivation, microorganisms rely on light energy to generate energy, whereas in heterotrophic cultivation, organic carbon sources are used for metabolism. Mixotrophic conditions combine autotrophic and heterotrophic models so that the cultured microorganisms have both a supply of inorganic carbon to be fixed via photosynthesis and some organic carbon source, such as glucose, glycerol, and acetate [97]. The microorganisms in mixotrophic cultures grow faster and can synthesize compounds through both autotrophic and heterotrophic pathways. Moreover, they have a reduced cost of light energy (compared with autotrophic culture) and organic compounds (compared with heterotrophic culture) [98].

### Modifications and improvement of the algal strains

The future feasibility of bioethanol as an alternative to fossil fuels will largely depend on its economic advantages. At present, microalgae biomass production does not permit commercial production of bioethanol [99]. The low carbohydrate content in algal biomass is a strong limitation. The amount of algal carbohydrate can be induced to reach a higher level than normal, either by controlling environment conditions or by introducing genetic modifications. Growing algal cells under certain stress conditions can alter certain biochemical pathways, leading to enhanced synthesis of carbohydrates [100, 101]. These stress conditions may be limitation of nutrients, such as nitrogen and phosphorus; change in light intensity, salinity of the growth media, or pH; or application of UV radiation. Regarding genetic modification, metabolic pathways inside the algal cell can be modified to increase the production of carbohydrates, lipids, and other important compounds of interest [102, 103]. It is important to select microalgae strains that allow easy, multidimensional modification of biochemical pathways. This novel approach uses new, powerful, rapidly evolving genetic engineering tools to identify and selectively modify the right genes.

Hence, efforts are increasing to develop carbohydrate-rich microalgae strains with the help of engineering approaches. Rapid development in genetics has made a number of transformation methods available, and successful trials encourage the use of genetic tools for a variety of purposes. Although the technology has not made satisfactory progress in the field of algal bioethanol yet, expectations for the near future are high.

## Pretreatment of algal biomass

**Fig. 3:**
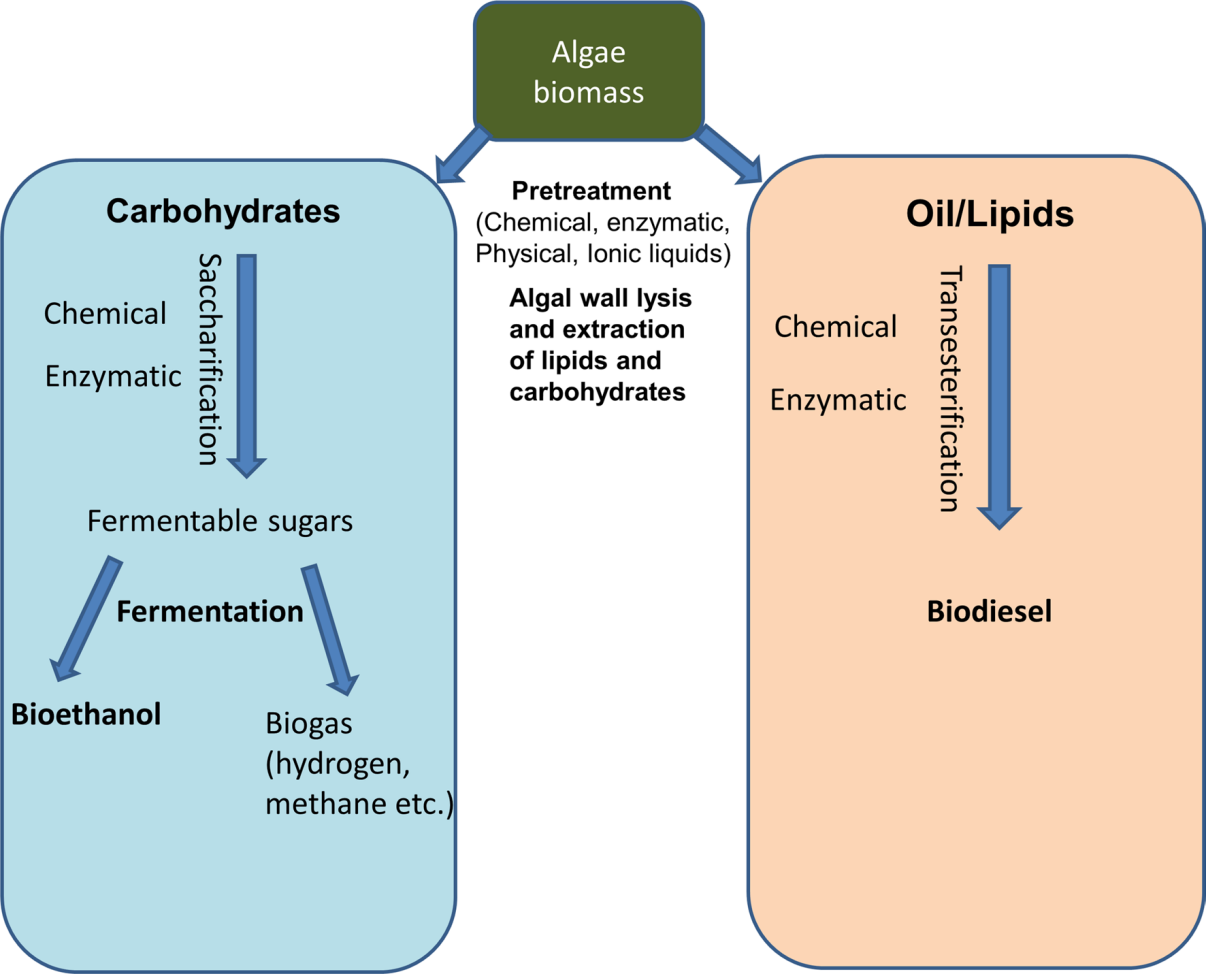
Pretreatment of microalgae biomass for biofuels production. Different types of biofuels can be produced depending on the raw materials used (carbohydrates and lipids) and the pretreatment prior to fermentation

Pretreatment of algal biomass involves the degradation or disruption of biomass to convert, accumulate, and process the carbohydrates and lipids it contains (Fig. 3). On a large scale, biomass pretreatment is a bottleneck and potentially costly step in biofuels production. Many different methods have been described for the pretreatment of algal biomass, but there is still no optimal, highly productive method. Researchers need to develop feasible and economical methods of biomass pretreatment for bioethanol production, optimized for different feedstocks.

Pretreatment of the algal biomass for fermentation involves lysis of algal cells to release the stored carbohydrates from inside the cells. The next step is the saccharification of the accumulated sugars to monomeric units, as fermenting microorganisms can only convert the fermentable forms of sugars (disaccharides and monosaccharides, usually hexoses and pentoses) into ethanol [104]. Algal biomass for bioethanol production is usually pretreated with acids and alkalis. Depending on the purpose of pretreatment, chemical, biological, thermochemical, or thermophysical methods are used, sometimes in combination [105, 106]. After lysis of the cells the macromolecules of sugars need saccharification which mean to break the α-(1 → 4), α-(1 → 6), β-(1 → 3) and β-(1 → 6) glycosidic linkages between the monomers to release the monomers units [107]. Lignocellulose present in the biomass impedes the pretreatment process, as this material is recalcitrant to conversion. The amount of lignocellulose also increases the cost of the whole pretreatment process. Hence, it is ideal if algal biomass has few or no lignocellulosic components present. This would make it economically more competitive than other feedstocks, such as terrestrial plants [108, 109]. Furthermore, the presence of cellulose in algal cell walls requires efficient degradation and conversation, approaches that also need to be optimized.

Algal biofuels technology needs to face the challenges of biomass harvesting and efficient pretreatment at low cost, with reduced emission of gases and high yields with scalable co-products. As different products are obtained from different source materials, pretreatments of biomass are often related to the products of interest. Thus, mechanical methods of pretreatment yield biodiesel, while enzymatic and chemical methods (including acidic hydrolysis) are used in bioethanol production, because fermentative bioethanol production requires degradation of cellulose, hemicellulose, and starch. In some cases, hydrothermal methods are also applied in combination with these. The cost of pretreating microalgae is far less than that of pretreating terrestrial plant biomass. Microalgae store photosynthetic products as cellulose, starch, and hemicellulose; some species also accumulate galactan. Algal cells are almost like lakes of lignin, which makes them a suitable and preferable feedstock for bioethanol production. Some of them contain other forms of carbohydrates that can be fermented to produce bioethanol and chemical feedstocks.

Hydrolysis with acids or enzymes can release fermentable sugars from algal carbohydrates for bioethanol production [8, 110, 111]. Acid hydrolysis is considered more effective than physical and thermal methods, but it requires high-temperature treatment (between 120 and 200 °C) to degrade algal biomass and convert the polymeric sugars of algal cells (e.g., starch and cellulose) to monomeric units. Strong acids, such as hydrochloric acid, sulfuric acid, and nitric acid, are commonly used. Acid strength, duration of treatment, and temperature considerably affect the yield of acid hydrolysis. Weak acids cannot successfully lyse the cells to extract the inner sugars, while excessively strong acids reduce sugars to furfural, thereby decreasing the final yield of monomeric sugars. In addition, furfural and its derivatives are toxic for the fermenting microorganisms, diminishing or even completely inhibiting fermentation [112, 113].

Some researchers have used mechanical methods, such as ultra-sonication, microwaves, and beating, to degrade algal biomass [114, 115]. Mechanical methods can be combined with chemical methods to reduce chemical waste and enhance the pretreatment process. They do not contaminate the environment, but they do impose higher costs, due to the consumption of energy [116, 117]. Enzymatic pretreatment of algal biomass is more productive and advantageous for fermentation processes [118, 119]. Enzymes such as amylases, cellulases, and invertases are common catalyzers that can hydrolyze a specific sugar substrate by selectively breaking the linkages between the units of the polysaccharide and releasing the monomer sugars. However, the high cost of enzymes makes this method exceedingly expensive for large-scale biomass pretreatment. An appropriate solution is to identify a microorganism that can overexpress the genes encoding these enzymes [120].

## Fermentation of the algal sugars to generate bioethanol

Fermentation is the biological process whereby sugars are converted to bioethanol by the action of fermenting microorganisms. Fermentable sugars, such as glucose, fructose, maltose, and rhamnose, are used as substrates by microorganisms in the fermentation process, which converts them to ethanol and CO_2_ [121, 122]. Several types of microorganisms, such as bacteria, yeasts, and fungi, have been identified for their potential for fermenting sugars to bioethanol [123–125]. The yield and quality of the bioethanol produced is strongly dependent on the fermentation process, which is affected by several factors, such as temperature, pH, oxygen, substrate concentration, and the fermenter organism used [126–130].

The algal sugars are excellent materials for bioethanol production. As mentioned above some species of microalgae are most adventitious for bioethanol production due to their higher sugars profile. However, the carbohydrates in algal biomass are mostly in polymeric form and need conversion to monomers unit to be readily fermented by microorganisms to produce bioethanol [131]. Veracious scientist reported various yields of fermentable sugars and ethanol from different species and strains of microalgae.

*Scenedesmus dimorphus* accumulated 53.7 w/w carbohydrate contents which on hydrolysis with sulfuric acids produced 80% fermentable sugars, indicating its feasibility for bioethanol production [132]. 11.7 g/l final ethanol yield was reported from Chlorella vulgaris [111]. Sivaramakrishnan et al. [133] recently reported 93% of ethanol yield from fermentation of *Scenedesmus* sp. derived sugars.

To scale up the fermentation process and define a commercialization strategy for bioethanol, the problems associated with alcoholic fermentation must be overcome. For bioethanol production to be viable, all the different types of sugars (pentoses and hexoses) must be converted to bioethanol. Fermenting microorganisms are substrate-dependent, so the substrate should be chosen carefully to maximize yields. Algae biomass contains different types of fermentable sugars, therefore combination of the fermenter microorganisms is preferable and effective, because different microorganisms have different rates of conversion for different substrates [134].

## Use of microalgae for food feed and bioproducts

The indigenous use of algae as food sources is an ancient practice. Many species of green algae have been utilized as food from ancient times [135]. Cultivation of microalgae started only a few decades ago, when it became clear that the fast-growing world population was likely to suffer a lack of protein-rich food stuffs [136]. Microalgae are an excellent source of food and other important bioproducts, such as natural antibiotics [137–139]. The world energy crisis in the 1970s led to the identification of algae as renewable and sustainable sources for biofuels production, prompting the exploration of microalgae as a new field of research for fuels and other valuable products [10, 140]. The first large-scale culture of the microalga Chlorella for commercial purposes was reported in Japan, in the 1960s [141]. Over the last few decades, algae culturing expanded to new fields, such as food and feed, biofuels, and biopharmaceuticals. Natural products in algal extracts are used in cosmetics and medicinal products [5]. According to one estimate, about 5000 metric tons of dry algal biomass processed for bioproducts generates US$ 1.25 × 109 each year [140].

Microalgae produce a wide range of other commercially important and valuable products. They produce vitamins, which elevates their importance as a nutritional food for people and animals [136, 142]. They also produce different types of medicinally important polysaccharides. Various species produce bioactive and commercially important pigments, such as chlorophyll, β-carotene and other carotenoids, phycobiliproteins, and astaxanthin. These pigments are crucial in therapies for tumorigenesis, neuronal disorders, and optical diseases. Microalgae are also rich sources of protein. Their production of essential amino acids increases their potential for use as protein-rich foods [143, 144]. Microalgae synthesize starch, cellulose, hemicelluloses, and other polysaccharides from simple monomeric sugars: basically, glucose. The higher amounts of carbohydrates in algal cells make them an important food source [144]. Microalgae also produce and accumulate large amounts of lipids, which vary among species and are affected by various factors [145]. Lipids in algal cells are present mainly in the form of glycerol, esterified sugars to different types of fatty acids (12–22 carbon atoms). Algal fatty acids have nutritional and medicinal applications. Most of the substances produced by microalgae have therapeutic effects. Therefore, a new area of research is extracting and identifying substances from microalgae and determining their biological and medicinal activities. Microalgae are becoming economical sources of natural substances for use as food and in cosmetics [146].

Pharmaceuticals on the market mainly consist of tablets or liquid forms of health-promoting substances, but several microalgae species are available as a supplement of various active substances in extract form, a new trend in the market. The microalgae market is growing due to the increasing demand for beneficial algal food and health products [147]. Polyunsaturated fatty acids produced by microalgae are important commercial products of high therapeutic value for cardiac diseases, asthma, and arthritis [148]. Many important microalgae products, such as eicosapentaenoic acid and docosahexaenoic acid (DHA), have been marketed by various biotechnological companies. Some species of microalgae produce protective substances against free radicals to prevent oxidative stress. These compounds are utilized as antioxidants in nutraceuticals and foods.

Researchers are taking a keen interest in algal substances with antioxidant properties that may be used in beverages and functional foods. These natural substances are highly important in pharmaceutical formulations for the treatment of free radicals and oxidative stress–associated diseases and complications. Blue-green microalgae (cyanobacteria) are rich in various pigment compounds that enhance the efficiency of light energy utilization (phycobiliproteins) and protect photosynthetic pigments from photo-oxidation (carotenoids). Currently, microalgae products with high nutritional value are available both in pure form as extracts, tablets, or capsules and as additives to several food products, such as candy bars, gums, pastas, and beverages [149]. These products are either used as nutrients or food coloring agents. Many microalgae strains, such as *Aphanizomenon flos*-*aquae, Chlorella, and Arthrospira*, are being cultured at a commercial scale for their high protein content and other health-promoting substances [143]. These products reportedly have important biological effects, such as anti-hyperglycemia and anti-hyperlipidemia, which are helpful in diabetes and obesity control because they affect the elevated serum glucose level.6

### Stress conditions

Although microalgae have many potential applications for biofuel and other useful products, 48 their production has not been fully commercialized because of several obstacles and challenges. Under normal conditions, microalgae may not produce important metabolites or produce them only in very small amounts. However, microalgae can be induced to synthesize these compounds by subjecting them to stressful conditions. This property of microalgae is considered very important for enhanced production of carbohydrates, lipids, astaxanthin, and other products [150, 151]. Stress conditions are unfavorable environmental factors, such as strong light, high salinity, high temperature, deprivation of nitrogen or other nutrients, short-term exposure to UV radiation, or a combination of these factors [152].

Astaxanthins are multifunctional carotenoids usually obtained from *Haematoccocus pluvialis.* This microalga synthesizes astaxanthin in response to environmental stress [153]. *Microcystis* sp. also synthesizes many important metabolites in response to unfavorable conditions, as a defense mechanism. *Microcystis aeruginosa* produces blooms in response to unfavorable environmental conditions, with excessive synthesis and release of toxic metabolites known as microcystins. Many researchers have reported that cyanobacteria produce compounds in response to environmental stress [154] Synthesis of microcystins and other metabolites by *M. aeruginosa* is greatly induced and influenced by light intensity, temperature, pH, and nutrients, such as carbon, nitrogen, and phosphorus [155].

### Significant compounds produced by microalgae

**Fig. 4:**
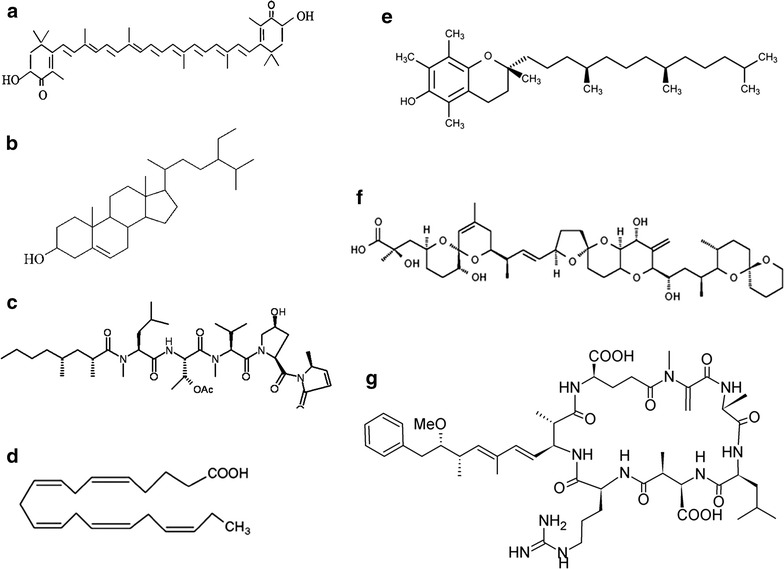
Some value-added compounds produced by microalgae. **a** Astaxanthin, a strong antioxidant produced by the microalga Haematococcus pluvialis. **b** β-Sitosterol, produced by various genera of micro algae. Different species of *Glaucocystophyte* produce β-sitosterol. **c** Microcolin-A, an immunosuppressive agent produced by the microalga *Lyngbya majuscule.* **d** Docosahexaenoic acid DHA produced by the engineered strain of diatom *Phaeodactylum tricornutum.* **e** Structure of vitamin E (tocopherols), the microalga *Haslea ostrearia* naturaly produce vitamin E. **f** Chemical structure of okadaic acid, an anti-fungal agent produced by some species of dinoflagellates. **g** Chemical structure of microcystin-LR, produced by the blooming *Microcystis aeruginosa*

Some of the highly valuable bioactive products isolated from microalgae are discussed below. Figure 4 shows their chemical structure.

#### Carotenoids

Carotenoids are important bio compounds having strong role in food, feed cosmetics and biopharma [156]. have Algae synthesize different types of pigments that possess important biological activities and thus are of great commercial interest. Among the most important are the phycobiliproteins, phycocyanin, phycoerythrin, β-carotene, lutein and astaxanthin [157]. Phycobiliprotein pigments are used in microscopy as fluorescent agents [158]. Phycocyanin and other pigments from red algae have antioxidant and anti-inflammatory effects; thus, they are used in food and cosmetic products [159, 160]. The microalga *Dunaliella salina* produces the carotenoid pigment β-carotene in quantities that represent about 10–14% of its dry mass [161]. β-carotene has a crucial role in vision and the immune system, due to its relation to vitamin A. Another important carotenoid pigment is astaxanthin, which is sold for 2500 US$/kg in the market. The microalga *Haematococcus. pluvialis* produce 4–5% astaxanthin per dry biomass [162]. Dried biomass of *Haematococcus pluvialis* has been commercialized as astaxanthin rich source. Because of their strong antioxidant activity, carotenoids are therapeutic in oxidative stress–related diseases and their complications, such as diabetes, aging, cancer, obesity, and stroke [163–165]. β-carotene and astaxanthin also have strong effects on the enzymatic antioxidant defense system by preventing oxidative stress through scavenging of free radicals [166]. β-Carotene protects membrane lipids from peroxidation, which is linked with many severe and lethal diseases, such as cancer, cardiovascular disease, Parkinson’s disease, and atherosclerosis [167–169]. The cis and trans forms of β-carotene are different isomers that confer the anticancer effect [170].

Many other compounds besides carotenoids have strong antioxidant activities, such as phenolic compounds and vitamins.

#### Sterols

Sterols produced by plants are called phytosterols. Microalga have a good contribution in the production of phytosterols and are considered potent and promising sources for the large-scale production. Some microalgae species have high levels of sterols. Microalgal sterols have some beneficial health effects like hypo-cholesterolemia, anticancer, anti-inflammatory and neurological diseases like Parkinson disease [171, 172]. Phytosterols used in pharmaceutical formulation for health benefits and nutraceuticals as food ingredients [173, 174]. Some microalgae species, such as those in the *Pavlova* and *Thalassiosira* genera, are rich in sterols [175, 176]. The microalga *Chaetoceros* has been reported to produce 27.7 μg sterols per gram of dry weight [177]. 40 different sterols have been identified in 100 different species of diatoms. These sterols are differed in chemical structures and some genera, e.g. *Amphora* produce distinctive types of sterols [178]. Recently the microalgae *annochloropsis* sp, *Pavlova lutheri*, *Tetrasellimis* sp have been screened to produce sterols with a net yield of 0.4–2.6%/dry weight) [179]. *Euglena gracilis* produce a mixture of sterols, 0.68–3.24 mg/g of dry biomass [180]. The major types of sterols reported in *Glaucocystophyte* are sitosterol, campesterol and stigmasterol, [181]. 24-Ethylcholesterol are mostly produced by cyanobacteria [182]. Dinoflagellates mostly produce 4α-methyl sterols and 24-propylidenecholesterol is mostly produced by *Pelagophyceae* [183, 184]. Synthesis of sterols is also influenced by a number of factors in microalgae affecting the final yield.

#### Proteins and enzymes

Some proteins, peptides, and amino acids have strong therapeutic effects on health or are necessary for cells and tissues to perform their normal activities. If the human body is unable to synthesize them, they must be obtained from an external source, usually food. Many species of microalgae produce higher quantities of various essential amino acids and proteins, which can be utilized in food and to protect against several diseases. Some species of microalgae can produce as much proteins as other rich sources of proteins, e.g. egg, meat and milk etc. [185]. Microalgae proteins have comparatively high nutritionals value. Microalgae produce 2.5–7.5 tons/Ha/year of proteins [186] the green microalga *Chlorella* is a rich source of different types of proteins, which have been marketed. Another protein-rich microalga is *Arthrospira*. Proteins from microalgae or plants cam reduce cholesterol levels by activating cholecystokinin. They also have important enzymatic effects [187]. *Lyngbya majuscula* produces microcolin-A, an immunosuppressive agent [188]. *Nostoc* produce the protein Cyanovirin which have been reported for its antiviral activities against HIV an influenza virus [189]. *Anabaena* and *Porphyridium* produce the enzyme SOD (superoxide dismutase), which protects against oxidative damages, while *Isochrysis galbana* produces the vital enzyme carbonic anhydrase, which plays a crucial role in converting CO_2_ into carbonic acid and bicarbonate. *M. aeruginosa* produces a variety of amino acids, including proline, serine, glycine, and valine.

#### Polyunsaturated fatty acids

Polyunsaturated fatty acids are important in tissue integrity and have beneficial health effects. Omega-3 and omega-6 fatty acids in particular are crucially important for humans, but the human body cannot produce these fatty acids. Thus, intake from external sources such as foods or cosmetics is essential. DHA, linoleic acid, eicosapentaenoic acid, arachidonic acid, and gamma-linolenic acid have been shown to suppress cholesterol levels, delay aging, guard membrane integrity, and prevent cardiovascular diseases [190, 191]. Many microalgae species (e.g. *Porphyridium cruentum*, *Arthrospira platensis, Odontella, I. galbana*) have been explored for their ability to synthesize these valuable fatty acids. *Pavlova lutheri* produces polyunsaturated fatty acids in large quantities, [192] while *A. platensis* produces and accumulates stigmasterol, sitosterol and γ-linolenic acid [177].

Eicosapentaenoic acid (EPA) and docosahexaenoic acid DHA are the medicinally important Omega-3 Polyunsaturated crucial in inflammatory diseases, heart problems, arthritis, asthma, and headache etc. [401]. EPA, DHA are produced by serval microalgae species sustainable and promising source and the only alternative to fish oils which are limited and unable to fulfill the required demands of EPA and DHA [193, 194]. Some efforts have been made recently to enhance the production of EPA and BHA by altering the metabolic pathways via genetic manipulation [195]. Most recently *Phaeodactylum tricornutum* attracted attentions as a potential source of EPA and DHA production [196–198]. The diatom *P. tricornutum* has been genetically sequenced and modified for enhance production of Omega-3 polyunsaturated fatty acids like EPA and DHA etc. It has been reported that the genetically engineered strain of *P. tricornutum* produce a maximum yield of 36.5 and 23.6% of DHA and EPA per total fatty acids of biomass indicating its feasibility for production of EPA and DHA at commercial scale [199].

However, commercial scale production of these significant and useful products such as EPA and DHA from microalgae need to overcome several hurdles and challenges which are responsible for its low product yields [200]. Up scaling required optimization in several areas, e.g. screening and selections of strains, culture development, products induction and extraction technologies. The algae growth and productions of Omega-3 Polyunsaturated fatty acids is greatly influence by carbon sources and light strength [201–203].

#### Vitamins

Microalgae are also rich sources of different vitamins. *Haslea ostrearia* is a rich source of vitamin E (tocopherols). *P. cruentum* produces high quantities of vitamins E and C, as well as β-carotene (vitamin A) [204]. The microalga *D. salina* readily produces vitamins A and E, pyridoxine, nicotinic acid thiamine, riboflavin, and biotin [205].

#### Toxic metabolites

Most microalgae species, especially cyanobacteria, produce a variety of toxic substances, generally called cyanotoxins. The most common examples are the microcystins produced by the blooming *Microcystis* species. *M. aeruginosa* is the dominant microalga of the bloom-secreting microcystins, which have been reported to be lethal for animals and humans. The bloom is also responsible for shellfish poisoning, due to the presence of toxins. Different types of microcystins have been identified. The microcystins are hepatocyclic peptides with a C20 amino acid chain, which determines the degree of toxicity [206]. Among all the microcystins produced by *M. aeruginosa*, microcystin LR is the most toxic and causes the death of animals and humans upon oral contact. Studies have shown that cyanobacterial toxins can treat tumors [207, 208].

Cyanotoxins are of great interest as environmental hazards and due to the chemistry of their toxicology [209]. They are broadly classified on the basis of (1) effects on vertebrates, divided into neurotoxins, hepatotoxins, and dermatotoxins; and (2) chemical structure as cyclic peptides, alkaloids, or lipopolysaccharides [210]. These toxic substances also have important antibacterial and antifungal activities [211, 212]. Other microalgae species have been explored for the production of toxic products. The diatom *Nitzschia pungens* produces domoic acid, which causes poisoning of shellfish [213]. *Gambierdiscus toxicus* produces gambieric acids, which have antifungal activities [214]. Karatungiols are active antimycotic agents produced by *Amphidinium*, which also have antiprotozoal activities [215].

## Biological activities of natural products from microalgae

### Antioxidant activity

Antioxidants are very important substances used by the human body to protect itself from the hazardous effects of free radicals. ROS (reactive oxygen species) and NOS (nitrogen reactive species) attack biomolecules like DNA, proteins, and membrane lipids, leading to many severe diseases including cancer, coronary arteries disease, obesity, diabetes, ischemic stroke, and Alzheimer disease [216].

Free radicals cause lipid peroxidation both in food lipids and biological membranes. Peroxidation results in various diseases and complications. In food materials, lipid peroxidation reduces shelf life and nutritional value. Antioxidants can prevent oxidative damage to cells and tissues by scavenging free radicals. The human body has its own enzymatic antioxidant system that prevents oxidative stress and protects the body from the hazardous effects of free radicals. However, when free radicals overcome the body’s natural antioxidants, oxidative stress occurs, which is one of the major causes of various dangerous and life-threatening diseases. In such cases, the uptake of external antioxidants is crucially important. Many natural antioxidant compounds have been reported. Compounds such as flavonoids, carotenoids, and vitamins like ascorbic acid and tocopherols have strong antioxidant activity.

In the pharmaceutical and food industries, several synthetic or natural antioxidants have been used to prevent oxidation and peroxidation processes [217]. As synthetic antioxidants have been shown to have side effects, natural antioxidants are sought after [218]. Recently, the exploration of natural antioxidants for nutraceuticals and pharmaceuticals industries has increased. Researchers are looking for antioxidants from natural sources, such as medicinal plants. Because of their huge potential for producing biologically active natural products, microalgae are one of the richest and most economical sources of natural compounds with strong antioxidants effects [219]. The antioxidant potential of these substances has been determined by various methods, including ABTS, DPPH radicals scavenging assay, ferric reducing potential, and metals chelating essays. Structural features such as a phytyl chain, a porphyrin ring, and conjugated double bonds are responsible for the antioxidant qualities [220]. Chlorophyll a and its metabolites produced by microalgae species are reported to have antioxidant activities [221]. as do most of the pigment metabolites of microalgae. The pigment fucoxanthin and its derivatives, such as auroxanthin, isolated from the microalga *Undaria pinnatifida*, have strong radical scavenging action [222]. Fucoxanthin is reported to have higher antioxidant effects than β-Carotene in tests of rat liver and plasma [223, 224]. The chemical structure of fucoxanthin shows two hydroxyl groups in a ring structure, which are considered the active moiety for free radical scavenging [225]. Phycobiliproteins (e.g., C-phycocyanin, R-phycoerythrin) are commercially used in the food and cosmetic industries as natural dyes [226]. Phycoerythrobilin, produced by some species of microalgae, has been shown to possess antioxidant activity [227]. Hence, the widespread existence of natural products with strong antioxidant activity increases the economical and nutritional potential of microalgae for the food, pharmaceutical, and nutraceutical industries.

### Anti-angiogenic, cytotoxic, and anticancer activities

Angiogenesis is the physiological process of developing new blood vessels from preexisting blood vessels. Angiogenesis proceeds rapidly during uterus development, embryogenesis, and wound healing. Angiogenesis breaks cell-to-cell contact and degrades the endothelium and extracellular matrix. The process involves the proliferation and migration of endothelial cells and formation of capillary tubes [228]. Although angiogenesis is a normal process, it may become pathological under certain conditions, such as cancer, atherosclerosis, arthritis, diabetic retinopathy, and ischemic stroke [229]. Pathologic angiogenesis promotes tumors and helps them to grow [230] Therefore, angiogenesis is considered the cause of tumor growth and expansion in cancer. Several activators and inhibitors are involved in the regulation of angiogenesis. The major antigenic factors and proteins are VEGF, PDGF, angiopoetin-1 angiopoietin-2, platelet-derived growth factor, interlukin-8, interlukin-8, bFGF, and angiotensin II [231, 232].

Many reports indicate the potential of natural products, including those produced by microalgae, to treat cancer and tumors by inhibiting angiogenesis. Fucoxanthin, found in many species of microalgae, significantly inhibits human blood cell proliferation and tube formation of HUVECs (human umbilical vein endothelial cells). Fucoxanthin and fucoxanthinol have been shown to inhibit the angiogenesis process in the aortic ring of rats by suppressing the growth of microvessels [233]. Some species of algae produce siphonaxanthin, which also possess antiangiogenic activity [234]. Fucoxanthin also has therapeutic effects for diabetes and induced the synthesis of arachidonic acid and DHA content in mouse livers [235]. It inhibits skin melanogenesis by negative regulation of the transcriptional factors involved [236]. Moreover, fucoxanthin has been shown to protect DNA from photooxidation [237]. Aerucyclamide is used in pharmaceutical products as an anti-plasmodial agent isolated from the blooming of *M. aeruginosa* [238]. Microalgae, particularly blue-green algae, are now being considered potential sources of active ingredients that can be utilized in the treatment of cancer. Many studies have shown the anti-cancer activity of these active products in the lab [239]. The mode of action and the mechanism of the activities may differ. Some of the microalgae-derived anti-cancer agents have been shown to induce apoptosis in tumorous cells by destroying the chromatin network, leading to cell death [240]. Cyanobacteria produce various metabolites by the ribosomal or non-ribosomal pathway. Most of these compounds are either peptides or alkaloids [241]. Peptides, including but not limited to those from cyanobacteria, tend to be toxic substances possessing strong cytotoxic activity. Cytotoxicity of these compounds is crucial in inducing apoptosis, which leads to cell death [242].

Many species of cyanobacteria (blue-green algae) produce apoptosis-inducing compounds. Apoptotic cells can be identified by their specific morphology, as they typically have a large cytoplasm and compressed organelles, with alterations of the chromatin materials. Extracts of *Synechocystis* sp. and *Synechococcus* sp. have been shown to drive HL-60 cells into apoptotic conditions. After treatment with extracts, cells express apoptotic markers, such as fragmentation of nucleus, shrinkage of cells, and release of apoptotic substances [240]. Similarly, *Lyngbya* sp. produce the glycoside biselyngbyaside, which can drive the mature osteoclasts into apoptotic conditions [243]. Extracts of Anabaena sp. have induced apoptosis in a leukemia myeloid cell line [244]. Some species of *Nostoc* produce cryptophycin, which is several hundred to a thousand times more active on cancer cells, such as human colorectal cancer, more effective than vinblastine or taxol [245]. The Oscillatoria boryana extract was active against human breast cancer [246]. *Microcystis* sp. in particular have great potential in the fields of toxicology and pharmacology, due to their production of bioactive metabolites and toxic substances with anti-cancer activity. The isolation of these compounds and determination of their biotechnological and toxicological applications continues to be studied by various researchers [247].

### Anti-obesity activity of algal products

Obesity is the overaccumulation of adipose tissues (fats) in the body [248]. Obesity is considered a multifactorial metabolic disorder linked to many complications and diseases, such as cancer, cardiovascular disease, diabetes mellitus, and aging [249]. The overgrowth of adipose tissue occurs because of adipogenesis, so obesity can be controlled by guarding the cells against adipogenesis [250]. Research has revealed various anti-hyperlidimic and fat-lowering agents from natural sources, such as medicinal plants. Microalgae are now being studied as potential sources of these products. ROS and NOS have also been reported to be involved in the progression of obesity. Thus, antioxidants can be used to control free radical–induced accumulation of fats. Fucoxanthin and fucoxanthinol can inhibit the differentiation of 3T3-L1 cells to adipocytes. Fucoxanthin and fucoxanthinol inhibit adipocyte differentiation by down-regulating peroxisome proliferator–activated receptor-c [251].

Okada et al. [252] reported that neoxanthin and fucoxanthin inhibited the accumulation of fats and stated that allenic and hydroxyl groups must be present to differentiate adipocytes. These compounds are reportedly crucial for lowering fat in high-fat mouse feeds [253] In obese mice, fucoxanthin suppresses adiposity by activating the mitochondrial protein UCP1 (Uncoupling protein1) in abdominal WAT [254] Fucoxanthin significantly reduced body fat in obese individuals in a clinical trial by Abidov et al. [255] *Cylindrotheca closterium* and *Phaeodactylum tricornutum* are the two microalgae species that produce fucoxanthin, [256] which shows potential as an anti-cancer, anti-oxidant, anti-obesity, anti-diabetic, and anti-inflammatory agent [257].

### Antimicrobial activities of microalgae metabolites

Bacteria and fungi are the major causes of severe diseases in plants and animals, including humans. They reduce crop yields and are the major causes of food spoilage. The widespread use of various antibiotics over the past few decades has given rise to increased resistance of microbes to antibiotics, making it necessary to search for new antimicrobial agents. Synthetic antibiotics have not achieved a satisfactory level of disease control due to side effects, high cost, and risk of generating severe resistant pathogenic strains. Therefore, researchers are searching for new natural antibiotics with a broad action spectrum from natural sources like plants and microorganisms. Natural products have comparatively fewer or no side effects.

Microalgae show a wide range of bioactive natural products that are effective, either in crude or purified form, as antioxidant, anti-cancer, and anti-microbial agents. The first reported antibacterial products in microalgae were in the green microalga *Chlorella*, which significantly inhibits the growth of both Gram-positive and Gram-negative bacteria [215]. Some microalgae also produce compounds that have antifungal activities [212]. Okadaic acid and ciguatoxin are effective antifungal agents produced by *Prorocentrum lima* and *G. toxicus*, respectively. Antimycotic activities have been reported for karatungiols, a group of compounds synthesized by the dinoflagellate *Amphidinium* [215]. *Chaetoceros lauderi* produces lipid metabolites that have been found to inhibit the growth of serval bacteria strains. *M. aeruginosa* is a rich source of several toxic metabolites that possess strong cytotoxic and antimicrobial effects. The crude extract of *M. aeruginosa* displays high antifungal and antibacterial activity [258]. *Dunaliella salina* also produces compounds that are active against several bacterial and fungus strains (e.g., *Staphylococcus aureus, Escherichia coli, Candida albicans, Psuedomonas aeruginosa, Aspergillus niger*) [259]. The extract of *D. salina* significantly inhibits the growth of *Klebsiella pneumoniae*. Substances synthesized by *Dunaliella primolecta* also showed antibacterial activity against *S. aureus* and against other bacterial strains [260].

## Conclusions

Microalgae are tiny factories and renewable, sustainable and economical sources of biofuels, bioactive medicinal products and food ingredients. Microalgae useful in mitigation of elevated CO_2_ level and treatment of waste water. Upgradation of algal fuel and bioproducts technology from pilot scale to commercial level is possible by overcoming the associated challenges and limitations. In this review we describe the extensive applications of the microalgae in bioenergy, nutraceutical and pharmaceutical industry, the associated challenges and limitations and how it can be overcome to make them feasible and viable for commercialization.
